# Factors associated with severe or fatal clinical manifestations of SARS‐CoV‐2 infection after receiving the third dose of vaccine

**DOI:** 10.1111/joim.13551

**Published:** 2022-08-09

**Authors:** Giovanni Corrao, Matteo Franchi, Danilo Cereda, Francesco Bortolan, Olivia Leoni, Jose Jara, Giuseppina Valenti, Giovanni Pavesi

**Affiliations:** ^1^ National Centre for Healthcare Research and Pharmacoepidemiology University of Milano‐Bicocca Milan Italy; ^2^ Unit of Biostatistics, Epidemiology and Public Health Department of Statistics and Quantitative Methods University of Milano‐Bicocca Milan Italy; ^3^ Directorate General for Health Lombardy Region Milan Italy; ^4^ ARIA S.p.a. Milan Italy

**Keywords:** COVID‐19, risk factors, SARS‐CoV‐2, severe disease, vaccine, vulnerability

## Abstract

**Background:**

Little is known about vulnerability to severe COVID‐19 illness after vaccination completion with three doses of vaccine against COVID‐19.

**Objectives:**

To identify individual features associated with increased risk of severe clinical manifestation of SARS‐CoV‐2 infections after receiving the third dose of vaccine against COVID‐19.

**Methods:**

We performed a nested case‐control study based on 3,360,116 citizens from Lombardy, Italy, aged 12 years or older who received the third dose of vaccine against COVID‐19 from 20 September through 31 December 2021. Individuals were followed from 14 days after vaccination completion until the occurrence of severe COVID‐19 illness, death unrelated to COVID‐19, emigration or 15 March 2022. For each case, controls were randomly selected to be 1:10 matched for the date of vaccination completion and municipality of residence. The association between candidate predictors and outcome was assessed through multivariable conditional logistic regression models.

**Results:**

During 12,538,330 person‐months of follow‐up, 5171 cases of severe illness occurred. As age increased, a trend towards increasing odds of severe illness was observed. Male gender was a significant risk factor. As the number of contacts with the Regional Health Service increased, a trend towards increasing odds of severe illness was observed. Having had a previous SARS‐CoV‐2 infection was a significant protective factor. Having received the Moderna vaccine significantly decreased the odds of severe illness. Significant higher odds were associated with 42 diseases/conditions. Odds ratios ranged from 1.23 (diseases of the musculoskeletal system) to 5.00 (autoimmune disease).

**Conclusions:**

This study provides useful insights for establishing priority in fourth‐dose vaccination programs.

## Graphical abstract

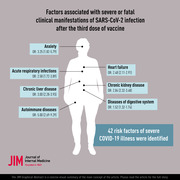

## Introduction

The emergence of novel SARS‐CoV‐2 variants [1] and the decreasing trend in the titres of antibodies in vaccinated individuals [2] have raised public health concerns regarding the efficacy and duration of protection induced by first‐generation vaccines [3]. The persistence of neutralizing antibodies and the degree of protection they confer remain largely unknown. Hence, understanding the risk of severe clinical manifestations of COVID‐19 after vaccination is completed provides an avenue to understanding the path to protection against COVID‐19 [4]. Finally, identifying predictors of post‐vaccine COVID‐19 disease is necessary for prioritizing interventions.

With the aim of shedding light on this field, we leveraged the integrated platform of the vaccination campaign of Lombardy, the largest Italian region including almost nine million candidates for vaccination—that is, beneficiaries of the Regional Health Service (RHS) aged 12 years or older. Using this observational database, we explored demographic and clinical characteristics associated with increased risk of severe COVID‐19 illness after receiving the third dose of vaccine.

## Materials and methods

In Lombardy, a population‐based platform was developed at the start of the vaccination campaign by means of record linking (i) the COVID‐19 vaccination registry (collecting the date, type and dose of vaccine dispensed), (ii) the registry of confirmed diagnosis of SARS‐CoV‐2 infection (collecting ascertained infections and hospital admissions, emergency‐room access and deaths due to COVID‐19) and (iii) the healthcare‐utilisation database (collecting various types of information, including causes of death, inpatient diagnoses supplied by public or private hospitals and outpatient drug dispensation). The different data can be interconnected because a single individual identification code is used in all databases. To preserve privacy, each identification code was deidentified automatically, with this inverse process being permitted only for the RHS at the request of judicial authorities. Further details of the healthcare databases used in the context of COVID‐19 in Lombardy have been reported [5, 6].

From 20 September until 31 December 2021, 3,388,667 beneficiaries of the RHS aged 12 years or older received their third dose of vaccine manufactured by Pfizer or Moderna. Citizens who experienced SARS‐CoV‐2 infection and/or COVID‐19 hospital admission or death within 14 days after receiving the vaccine were excluded. The remaining citizens entered the study cohort and were followed from 14 days after vaccination completion (under the assumption that immune coverage is achieved 2 weeks after receiving the vaccine [7]) until the outcome occurrence (see next paragraph), death from a cause different from COVID‐19, emigration or 15 March 2022, whichever occurred earliest.

A nested case‐control design was adopted by listing the cases of patients who, during follow‐up, experienced the first occurrence of severe or fatal clinical manifestations of SARS‐CoV‐2 infection—that is, hospital admission, including those in an intensive care unit, or death from COVID‐19—confirmed by a positive nasopharyngeal swab. The case series was denoted as ‘severe illness cases,’ and the date of outcome occurrence was denoted as ‘index date’.

For each patient in the severe illness case series, 10 controls were randomly selected from the study cohort among those who had not yet experienced the outcome at the index date, to be matched 1:10 for the date of vaccination completion and municipality of residence.

The following information was retrieved for each case and control: gender, age at cohort entry, previous occurrence of infection from SARS‐CoV‐2, type of vaccine received and medical pathway traced from contacts with the RHS during 2018 and 2019. The latter comprised categories of the number of contacts with the RHS, and the presence/absence of 59 diseases/conditions (candidate predictors) traced through hospital admissions and drug prescriptions. Details on the use of healthcare‐utilisation databases for tracing the conditions that each beneficiary of the RHS suffers from are reported elsewhere [8, 9, 10]. The list of candidate predictors included practically all nosological categories and was prepared taking into consideration morbidity and mortality predictors reported by selected systematic reviews and meta‐analyses [11, 12, 13, 14]. The list of candidate predictors and the corresponding codes are reported in the Supplementary Table.

With the aim of investigating the strength of association between the factors reported above and the odds of experiencing the outcome of interest, conditional logistic regression was fitted by including all the covariates in a unique model. Subsequently, with the aim of investigating the association between each single candidate predictor and the outcome of interest, conditional logistic regression was fitted by including one condition—which was included in the model as a dichotomous variable, with a value of 1 or 0 according to whether the specific condition was or was not recorded during the years 2018 and 2019—while adjusting for the covariates considered above. Only conditions affecting at least 10 cases of severe illness were included in this analysis. Finally, in order to measure the impact of the selected conditions on the risk of severe or fatal clinical manifestations of SARS‐CoV‐2 infection, the population attributable fraction was calculated as $\frac{p\times (\mathrm{OR}-1)}{p\times (\mathrm{OR}-1)+1}$, where ‘p’ represents the prevalence of exposure to a given disease/condition in the overall population [15].

## Results

The 3,360,116 citizens included in the study cohort had a mean age of 60.9 years (standard deviation 17.8 years), 45.8% were male and 55.7% received the Moderna vaccine. The cohort members accumulated 12,538,330 person‐months of observation (on average, almost 3.7 months each) and generated 5171 severe illnesses, corresponding to an incidence rate of 4.1 cases per 10,000 person‐months (95% confidence interval: 4.0–4.2 per 10,000 person‐months). Of these, 4917 cases were matched with 49,170 controls.

**Fig. 1:**
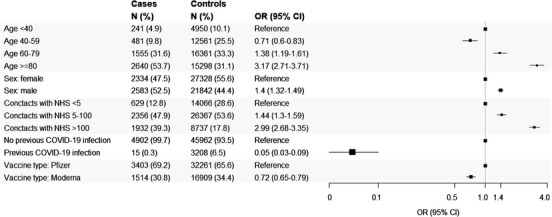
Forest plots depicting the association between selected features of the study cohort (citizens who completed scheduled vaccination plan with three doses) and the odds of severe COVID‐19 illness. Note: The analysis included 4917 patients who, starting from at least 14 days after completing the scheduled vaccination (with three doses), experienced COVID‐19 hospital admission, including those in an intensive care unit, or death, and 49,170 controls randomly selected to be 1:10 matched for the date of vaccination completion and municipality of residence, and for not having yet experienced severe illness on the date on which the corresponding case experienced it (index date). The number of cases and controls and the corresponding column percentage are reported for each feature. Conditional logistic regression models including all the considered features as covariates were fitted for estimating odds ratios and corresponding 95% confidence interval.

Risk factors of post‐vaccine severe COVID‐19 illness are pictured in Fig. 1. Male gender was a significant risk factor. The lowest value in odds ratio was reached for the age of 40–59 years, followed by increasing values, which reached the highest peak for ultraoctogenarians. As the number of contacts with the RHS increased, a trend towards increasing odds was observed. Having had a previous SARS‐CoV‐2 infection was a strong protective factor. There was statistical evidence that the Moderna vaccine conferred higher protection than Pfizer.

**Fig. 2:**
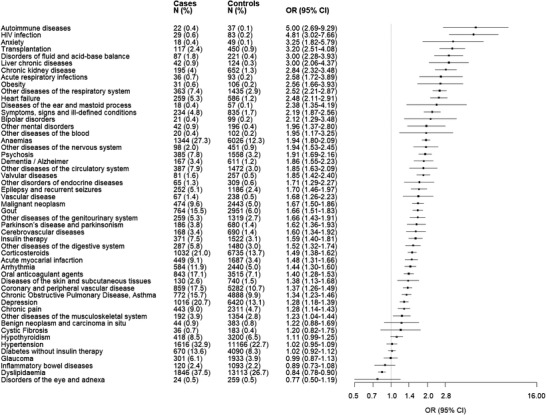
Forest plots depicting the association between 51 diseases/conditions the members of the study cohort (citizens who completed the scheduled vaccination plan) suffered from and the odds of severe COVID‐19 illness. The 51 diseases/conditions are sorted for decreasing values of the observed association strength. Note: The analysis included 4917 patients who, starting from at least 14 days after completing the scheduled vaccination (with three doses), experienced COVID‐19 hospital admission, including those in an intensive care unit, or death, and 49,710 controls randomly selected to be 1:10 matched for the date of vaccination completion and municipality of residence, and for not having yet experienced severe illness on the date on which the corresponding case experienced it (index date). The figure reports the number of cases and controls, and the corresponding percentages, exposed to 51 conditions. For each condition, the adjusted odds ratio (point estimate of the association strength) and the corresponding 95% confidence interval was estimated by fitting logistic regression models, including that condition while adjusting for the features listed in Fig. 1. The size of each box (point estimate of each condition) reflects the estimate's precision. The 51 conditions considered for this analysis have been selected from the 59 candidate predictors (Supplementary Table) because at least 10 patients suffered from it. By accepting a 5% one‐side first type error, the sample size reached by this analysis allows for appreciating an odds ratio ≥2.08 with 80% power, even for rare conditions/diseases (i.e., for predictors that occur in only 0.2% of controls).

The strength of association between the candidate predictors and severe illness is pictured in the forest plot of Fig. 2. Significant higher odds were associated with 42 diseases/conditions, that is, 71% of the investigated factors. Odds ratios ranged from 1.23 (diseases of the musculoskeletal system) to 5.00 (autoimmune diseases). Figures S1 and S2 show the unadjusted estimates of the association between candidate predictors and severe COVID‐19 illness. The prevalence of cohort members (i.e., citizens from Lombardy who received three vaccine doses) suffering from at least one disease/condition showing an odds ratio higher than 3, 2 or 1.5, or any condition showing statistical evidence of being associated with the considered outcome were 42,525 (1.3%), 163,374 (4.9%), 867,987 (25.8%) and 1,608,440 (47.9%), respectively.

It should be stressed, however, that some predictors that were able to strongly increase the risk of the considered outcome were also characterized by high prevalence (among controls), making the corresponding condition more relevant for its public health implications. Table 1 gives another ranking system based on population attributable fraction—that is, on the proportion of outcomes (severe/fatal clinical manifestations of SARS‐CoV‐2 infections) that may be avoided by protecting patients suffering from the corresponding condition.

**Table 1:** List of diseases/conditions associated with the risk of experiencing post‐vaccine severe or fatal clinical manifestation of SARS‐CoV‐2 infection according to population attributable fraction

Disease/Condition	Population attributable fraction (%)
Anaemias	10.3
Use of corticosteroids	6.3
Other diseases of the respiratory system	4.2
Coronary and peripheral vascular disease	3.8
Gout	3.8
Depression	3.5
Chronic obstructive pulmonary diseases, asthma	3.3
Malignant neoplasm	3.2
Psychosis	2.8
Use of oral anticoagulant agents	2.8
Other diseases of the circulatory system	2.5
Chronic kidney disease	2.4
Arrhythmia	2.1
Symptoms, signs and ill‐defined conditions	2.0
Transplantation	2.0
Insulin therapy	1.8
Other diseases of the genitourinary system	1.7
Heart failure	1.7
Epilepsy and recurrent seizures	1.7
Acute myocardial infarction	1.6
Other diseases of the digestive system	1.5
Chronic pain	1.3
Dementia/Alzheimer	1.1
Disorders of fluid and acid–base balance	0.9
Other diseases of the nervous system	0.9
Parkinson's disease and Parkinsonism	0.9
Cerebrovascular diseases	0.8
HIV infection	0.6
Other diseases of the musculoskeletal system	0.6
Diseases of the skin and subcutaneous tissues	0.6
Liver chronic disease	0.5
Other disorders of the endocrine system	0.4
Valvular diseases	0.4
Other mental disorders	0.4
Obesity	0.3
Vascular diseases	0.3
Autoimmune diseases	0.3
Acute respiratory infection	0.3
Bipolar disorders	0.2
Anxiety	0.2
Other diseases of the blood	0.2
Diseases of the ear and mastoid process	0.2

## Discussion

The current study, based on real‐world data from more than three million people who received three doses of vaccine against COVID‐19, identifies an extensive set of factors that increase the risk of post‐vaccine severe COVID‐19 illness. Factors such as old age, male gender, frequent contacts with the RHS and diseases/conditions affecting practically almost all organs and systems significantly increased the risk of severe COVID‐19 illness. These results provide useful insights for establishing priority in fourth‐dose vaccination programs, as well as in effective treatments. The lower odds of severe COVID‐19 illness among individuals aged 40–59 years as compared to those aged <40 years is an unexpected result, and the possible reasons for this finding are unknown to the authors.

Our study provides the following additional results: (1) The observed higher risk of severe illness among males is a widely expected finding. Consistently, it has been reported that despite the number and age of males and females with SARS‐CoV‐2 infection being comparable, males tend to display more severe disease [16, 17, 18]. Although several factors have been speculated to account for the disparity—including differences in biology, behaviour, occupation and immune response [19, 20]—the underlying mechanisms are still unclear [16]. (2) Having received the vaccine manufactured by Pfizer offered lower protection against severe post‐vaccine disease than having received the one by Moderna. (3) Our study provides further evidence that the risk of post‐vaccine COVID‐19 disease is strongly reduced among individuals who already experienced SARS‐CoV‐2 infection as compared to naïve individuals. With respect to the available evidence, however, our study goes beyond the comparison of immune response in recovered and naïve individuals [21, 22, 23, 24, 25, 26, 27, 28], extending such evidence to the protective action of previous infection against the post‐vaccine clinical manifestation of the infection [29]. (4) Finally, several conditions and diseases from which vaccinated citizens suffered strongly affected their risk of post‐vaccine severe illness. This confirms the now‐established notion that alterations of the structure and function of virtually all organs and systems of the body may adversely affect resistance to the COVID‐19 disease [11].

The present study has several points of strength. One, the study provides the largest and most robust available evidence of the post‐vaccine risk factors of severe/fatal COVID‐19 disease. Two, the study is based on a very large population and included all ages eligible for COVID‐19 vaccination. This allowed a large accumulation of person‐months, which means that although post‐vaccine cases of severe illness are very rare events, the study was sufficiently powered to address its primary goal.

The limitations are that the predictors of COVID‐19 we searched for are restricted to those routinely collected and available in the administrative databases, that is, hospital admissions and drugs dispensed. In addition, our system for tracking diseases did not capture the severity of the associated comorbidities. Furthermore, health services and treatments supplied by private providers were not captured by our analysis. Moreover, misdiagnosis—due to poor accuracy in reporting diagnoses and comorbidities—and upcoding in hospital records—sometimes in pursuit of higher reimbursements—might have led to underestimating the prevalence of patients affected by the considered conditions. By assuming that the misclassification error is the same between cases and controls (i.e., nondifferential misclassification), the observed odds ratios may be underestimated [30]. Finally, outcome misclassification may have affected the study because some patients with severe symptoms might have been treated at home.

## Conclusions

By using a large population‐based platform notable for monitoring the trend and impact of the vaccine campaign in the largest Italian region, we identified an extensive set of factors that increase the post‐vaccine risk of severe COVID‐19 illness. Factors such as old age, male gender, frequent contact with the RHS and diseases/conditions belonging to all the considered nosological categories strongly increased the risk of severe COVID‐19 illness. This suggests that post‐vaccine vulnerability to severe clinical manifestations of SARS‐CoV‐2 infection may mainly be affected by clinical frailty, possibly due to comorbidities, rather than to specific disorders [11]. Our study confirms that severe COVID‐19 illness may occur after the vaccination cycle is completed, although with low incidence. These findings would support efforts to maximize both vaccine uptake with three doses and individual protection measures. Clinicians and policymakers can use our results for prioritizing interventions, while researchers can utilize our findings to develop prognostic models that could eventually facilitate decision making.

## Conflict of interest

Giovanni Corrao received research support from the European Community (EC), the Italian Agency of Drugs (AIFA) and the Italian Ministry for University and Research (MIUR). He took part in a variety of projects that were funded by pharmaceutical companies (i.e., Novartis, GSK, Roche, AMGEN and BMS). He also received honoraria as a member of the advisory board to Roche.

## Author contributions

Giovanni Corrao: Conceptualization; Methodology; Project administration; Supervision; Writing – original draft. Matteo Franchi: Conceptualization; Methodology; Project administration; Supervision; Writing – original draft. Danilo Cereda: Conceptualization; Writing – review and editing. Francesco Bortolan: Conceptualization; Resources; Writing – review and editing. Olivia Leoni: Conceptualization; Writing – review and editing. Jose Jara: Formal analysis; Writing – review and editing. Giuseppina Valenti: Conceptualization; Writing – review and editing. Giovanni Pavesi: Conceptualization; Resources; Writing – review and editing.

## Supporting information




**Figure S1**. Forest plots depicting the unadjusted association between selected features of the study cohort (citizens who completed scheduled vaccination plan with three doses) and the odds of severe COVID‐19 illness.

**Figure S2**. Forest plots depicting the unadjusted association between 51 diseases/conditions the members of the study cohort (citizens who completed scheduled vaccination plan) suffered from and the odds of severe COVID‐19 illness. The 51 diseases/conditions are sorted for decreasing values of the observed association strength.

**Table S1**. List of candidate conditions for predicting the risk of experiencing post‐vaccine severe or fatal clinical manifestation of SARS‐CoV‐2 infection.
