# Supplementary figures and images for: Factors associated with severe or fatal clinical manifestations of SARS‐CoV‐2 infection after receiving the third dose of vaccine
Source: J Intern Med. 2022 Aug 9:10.1111/joim.13551. Online ahead of print. doi: 10.1111/joim.13551 (PMC9539163; doi:10.1111/joim.13551)

## Slide 1
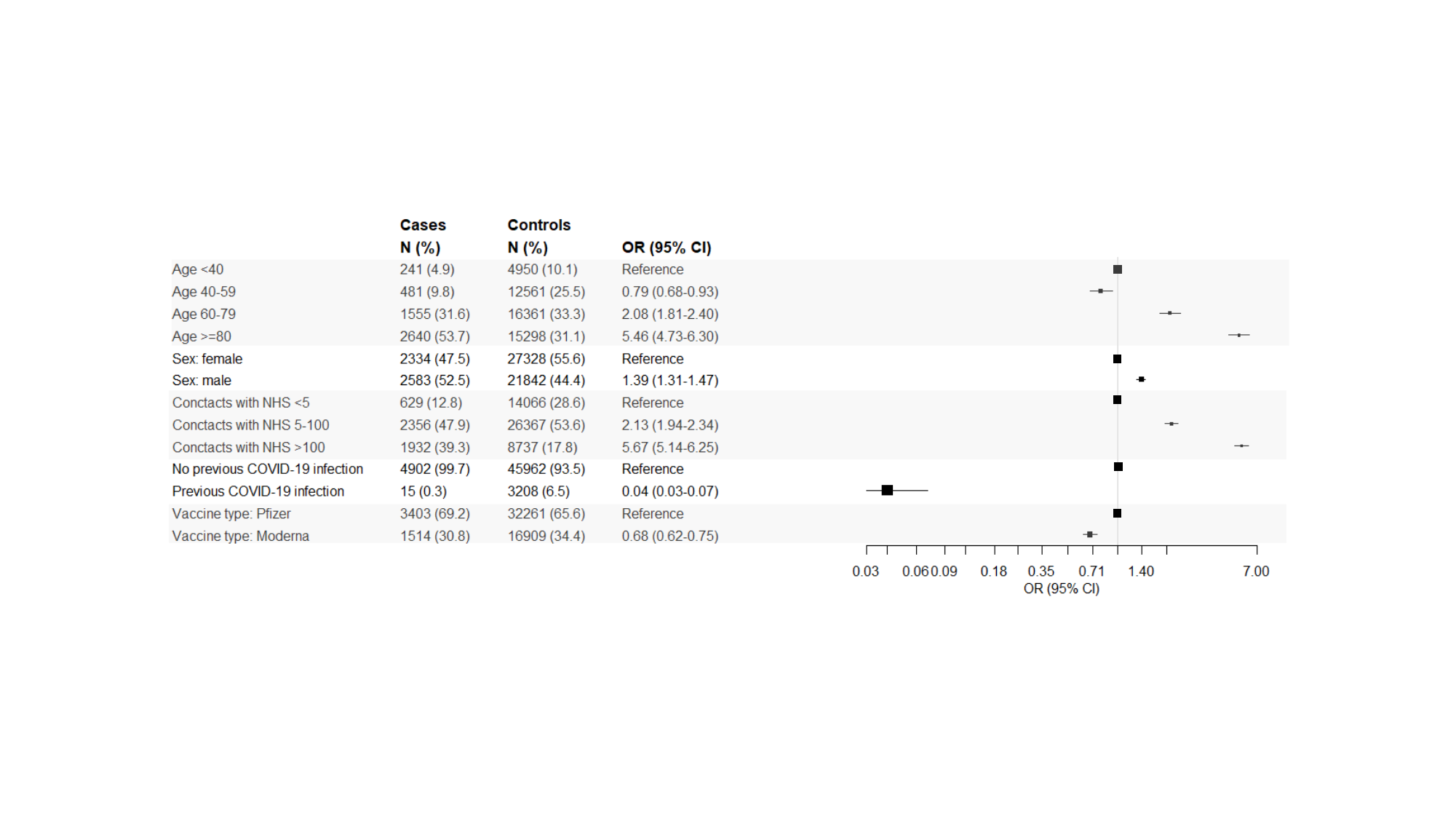

Supplement: Supplementary file 1 — Figure S1. Forest plots depicting the unadjusted association between selected features of the study cohort (citizens who completed scheduled vaccination plan with three doses) and the odds of severe COVID‐19 illness. [file JOIM-9999-0-s003.pptx]

## Slide 1
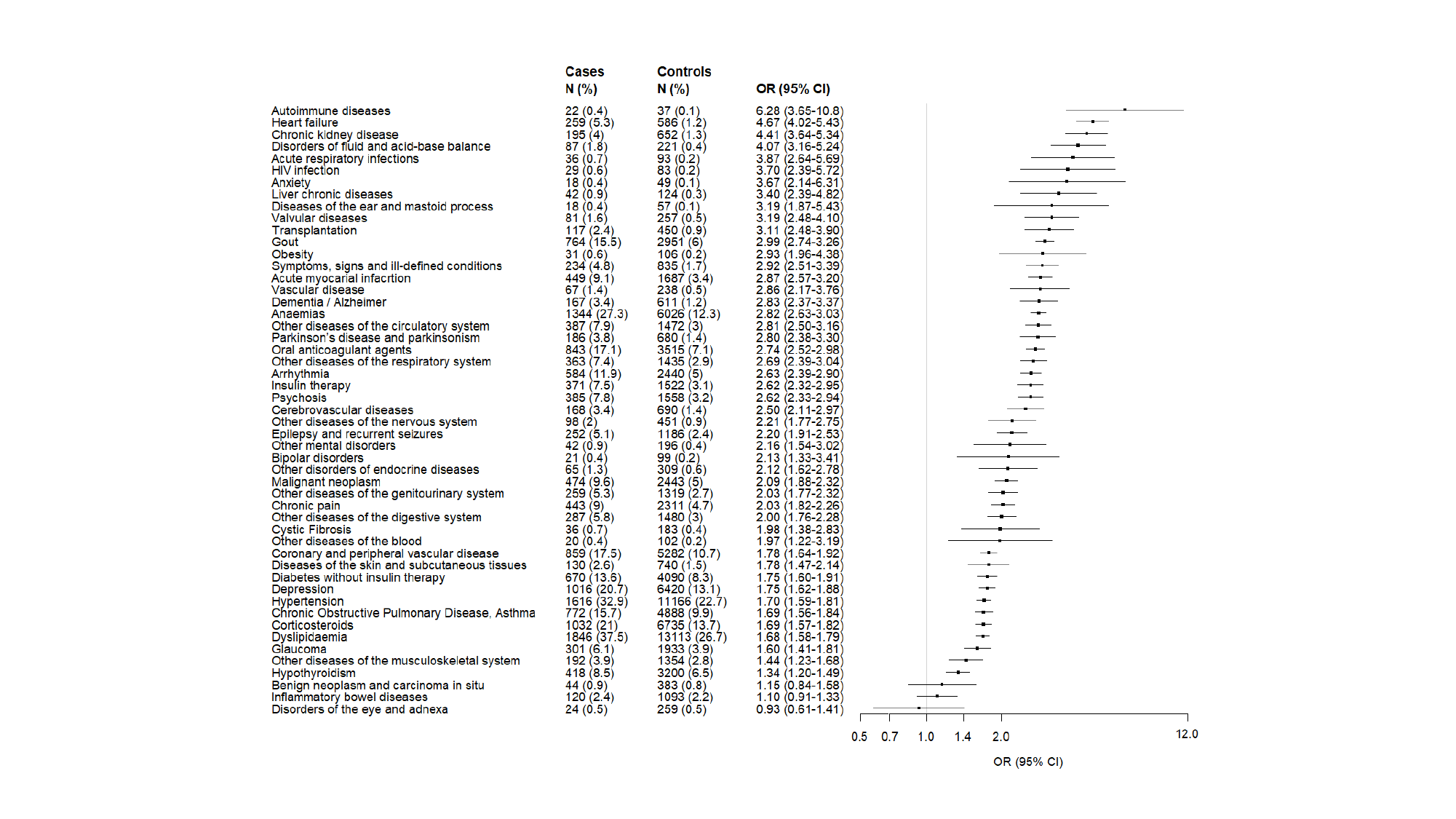

Supplement: Supplementary file 2 — Figure S2. Forest plots depicting the unadjusted association between 51 diseases/conditions the members of the study cohort (citizens who completed scheduled vaccination plan) suffered from and the odds of severe COVID‐19 illness. The 51 diseases/conditions are sorted for decreasing values of the observed association strength. [file JOIM-9999-0-s002.pptx]
