# Supplementary material for: Factors associated with severe or fatal clinical manifestations of SARS‐CoV‐2 infection after receiving the third dose of vaccine
Source: J Intern Med. 2022 Aug 9:10.1111/joim.13551. Online ahead of print. doi: 10.1111/joim.13551 (PMC9539163; doi:10.1111/joim.13551)
Supplement: Supplementary file 3 — Table S1. List of candidate conditions for predicting the risk of experiencing post‐vaccine severe or fatal clinical manifestation of SARS‐CoV‐2 infection. [file JOIM-9999-0-s001.docx]

**Supplementary Table S1. List of candidate conditions for predicting the risk of experiencing post-vaccine severe or fatal clinical manifestation of SARS-CoV-2 infection**

|  | **#** | **Disease / condition** | **ICD-9 CM** | **ATC** |
| --- | --- | --- | --- | --- |
| Infectious and parasitic diseases | 1 | HIV infection | 042.x, V08 | J05AB14, J05AE, J05AF01, J05AF02, J05AF04, J05AF05, J05AF06, J05AF09. J05AG, J05AR, J05AX07, J05AX08, J05AX09, J05AX12 |
|  | 2 | Tuberculosis and Other infectious and parasitic diseases | 010.x - 018.x, 001.x-009.x, 020.x-027.x, 030.x-0.41.x, 045.x–057.x, 060.x-066.x, 070.x-088.x, 090.x-104.x, 110.x-118.x, 120.x-139.x | J04AB |
| Neoplasms | 3 | Solid malignancies and Neoplasm of lymphatic and haematopoietic tissue | 140.x-165.x, 170.x-176.x, 179.x-199.x, V58.0, 92.2, 200.x-208-x | L01, L03AC, L02BA01, L02BA02, L02BG02, L02BG03, L02BG04, L02BG06, L02BB01, L02BB03, L02AE02, L02AE04, L02AB01 |
|  | 4 | Benign neoplasm and carcinoma in situ | 210.x-234.x |  |
| Endocrine, nutritional and metabolic diseases, and immunity disorders | 5 | Hypothyroidism | 243, 244.x | H03A, H03B |
|  | 6 | Diabetes without insulin therapy | 250.x, 348.0x, 357.2, 362.0, 366.41 | A10B |
|  | 7 | Insulin therapy |  | A10A |
|  | 8 | Dyslipidaemia | 272.2, 272.4 | C10 |
|  | 9 | Obesity | 278.0x |  |
|  | 10 | Weight loss | 260-263.x |  |
|  | 11 | Disorders of fluid, electrolyte, and acid-base balance | 276.x |  |
|  | 12 | Gout | 274.x | M04AC01, M04AA, M04AB |
|  | 13 | Other disorders of endocrine, nutritional and metabolic diseases | 240.x-242.x, 245.x, 246.x, 249.x, 251.x, 252.8, 252.9, 253.x-259.x, 264.x-269.x, 270.x, 271.x, 272.0, 272.1, 272.3, 272.5-272.9, 273.x, 275.x, 277.x, 278.1-278.8 (except 277.0) |  |
|  | 14 | Disorders involving the immune mechanisms | 279.x |  |
| Diseases of the blood and blood-forming organs | 15 | Coagulation defects | 286.x | B02B |
|  | 16 | Autoimmune haemolytic anaemias, Other anaemias, Anaemias only tracked from drug therapy | 280.x-282.x, 283.1-283.9, 284.x-285.x | B03A, B03B, B03XA01, L03AA |
|  | 17 | Other diseases of the blood and blood-forming organs | 287.x-289.x |  |
| Mental disorders | 18 | Dementia / Alzheimer | 290.0-290.4x, 331.0x | N06DA, N06DX01 |
|  | 19 | Psychosis | 295.x, 297.x, 298.2-298.9, 299.1x | N05AD, N05AA, N05AB, N05AC, N05AX, N05AE, N05AF, N05AG N05AH, N05AL |
|  | 20 | Depression | 296.2, 296.3, 296.82, 298.0, 300.4, 301.12, 309.0x, 309.1x, 311.x | N06A |
|  | 21 | Bipolar disorders | 296.0x, 296.1x, 296.4x, 296.5x, 296.6x, 296.7x, 296.80, 296.81, 296.89, 296.9x, 298.1x | N05AN |
|  | 22 | Alcohol abuse | 291.1, 291.2, 291.5, 291.8x, 291.9, 303.9, 305.0x, V11.3x | N07BB01 |
|  | 23 | Drug addition | 292.0x, 292.82-292.89, 292.9x, 304.x, 305.2x-305.9x | N07BB04 |
|  | 24 | Anxiety | 300.0x | N05BA, N05BB01, N05CD, N05BC01, N05BC51, N05BX, N05CF, N05CX01, N06BX |
|  | 25 | Other mental disorders | 290.8, 290.9, 291.0, 291.3, 291.4, 292.1x, 292.2, 292.81, 293.x, 294.x, 299.0x, 299.8x, 299.9x, 300.0x-300.2x, 300.3, 300.5-300.9, 301.0, 301.10, 301.11, 301.2x-301.9x, 302.x, 303.x, 305.1, 306.x-308.x, 309.2x-309.4x, 310.x, 312.x-319.x |  |
| Diseases of the nervous system and sense organs | 26 | Parkinson’s disease and parkinsonism | 332.x | N04 |
|  | 27 | Multiple sclerosis | 340 | L03AB07, L03AB08, L04AA23, L04AA27, L03AX13, L04AA31, L04AA34, L03AB13, L04AX07 |
|  | 28 | Epilepsy and recurrent seizures | 345.x | N03AF01, N03AB02, N03AA02, N03AA03, N03AA04, N03AE01, N03AD01, N03AG01, N05BA09, N03AG04, N03AX10, N03AG06, N03AF02, N03AX14, N03AX15 |
|  | 29 | Glaucoma | 365.x | S01E |
|  | 30 | Disorders of the eye and adnexa | 360.x-379.x (except 365.x) |  |
|  | 31 | Diseases of the ear and mastoid process | 380.x-389.x |  |
|  | 32 | Other diseases of the nervous system and sense organs | 320.x-326.x, 330.x-331.x, 333.x-337.x, 340.x-344.x, 346.x-359.x |  |
| Diseases of the circulatory system | 33 | Ischaemic Heart Disease/Angina | 410.x – 414 | C01DA, C01DX |
|  | 34 | Heart failure | 398.91, 402.11, 402.91, 404.11, 404.13, 404.91, 404.93, 428.x |  |
|  | 35 | Arrhythmia | 426.10, 426.11, 426.13, 426.20-426.53, 426.60-426.89, 427.0, 427.2, 427.31, 427.60,427.9, 785.0x, V45.0x, V53.3x | C01BA, C01BC, C01BD |
|  | 36 | Valvular diseases | 093.20-093.24, 394.0x-397.1x, 424.00-424.91, 746.3x-746.6x, V42.2x, V43.3x |  |
|  | 37 | Vascular diseases | 440.x, 441.2, 441.4, 441.7, 441.9, 443.1x-443.9x, 447.1, 557.1x, 557.9x, 785.4x, V43.4x |  |
|  | 38 | Cerebrovascular diseases | 430.x-438.x |  |
|  | 39 | Hypertension | 401.x-405.x | C03AA, C03AB, C03AH, C03AX01, C02CA04, C03BA02, C03BA03, C03BA04, C03BA05, C03BA07, C03BA08, C03BA09, C03BA10, C03BA11, C03DB01, C03DB02, C03EA, C09BA02, C09BA03, C09BA04, C09BA05, C09BA06, C09BA07, C09BA08, C09BA09, C09BB, C09DB, C09DA01, C09DA02, C09DA03, C09DA04, C09DA06, C09DA07, C09DA08, C02AB01, C02AB02, C02AC01, C02AC02, C02AC04, C02AC05, C02DB02, C02DB03, C02DB04, C02DC01, C02DD01, C02DG01, C02KA01, C02KB01, C02KC01, C02KD01, C02KX01, C09XA |
|  | 40 | Coronary and peripheral vascular disease |  | B01AB, B01AX01, B01AD10, B01AD12, C04AD03, B01AC05 |
|  | 41 | Oral anticoagulant agents |  | B01AA, B01AE, B01AF |
|  | 42 | Other diseases of the circulatory system | 390.x-392.x, 393, 397.9, 398.90, 398.99, 411.8x, 412.x-417x, 420.x-423.x, 424.99, 425.x, 426.0, 426.12, 426.54, 426.9, 427.1, 427.32, 427.4x, 427.5, 427.61, 427.69, 427.8x, 429.x, 441.0x, 441.1, 441.3, 441.5, 441.6, 442.x, 443.0, 444.x-446.x, 447.0, 447.2-447.9, 448.x 451.x-459.x |  |
| Diseases of the respiratory system | 43 | Chronic Obstructive Pulmonary Disease, Asthma, Chronic respiratory disease only tracked from drug therapy | 490-492.x, 493.x, 494.x, 496 | R03AA, R03AB, R03AC, R03DA, R03DB, R03DA20, R01AC01, R03BC01, R01AC51, S01GX01, S01GX51, R03BA |
|  | 44 | Acute respiratory infections | 460-466.x |  |
|  | 45 | Cystic Fibrosis | 277.0 | R05CB, R05FB01, R05FA01, A09AA02, R07AX02, R07AX30, R07AX31 |
|  | 46 | Other diseases of the respiratory system | 470.x-478.x, 480.x-487.x, 495.x, 500.x-508.x, 510.x-519.x |  |
| Diseases of the digestive system | 47 | Liver cirrhosis and other liver chronic diseases | 571.x, 573.x | J05AP08, J05AP09, J05AP51, J05AP53, J05AP54, J05AP55, J05AP56, J05AP57, B05AA01 |
|  | 48 | Inflammatory bowel diseases (Ulcerative colitis and Chron’s disease) | 555.x-556.x | A07EC01, A07EC02, A07EC03, A07EC04 |
|  | 49 | Chronic and acute pancreatitis | 577.0-577.1 |  |
|  | 50 | Other diseases of the digestive system | 520.x-553.x, 557.x-570, 572.x, 574.x-576.x, 577.2-577.9, 578.x, 579.x |  |
| Diseases of the genitourinary system | 51 | Chronic kidney disease | 585, V45.1, V56.x, V03AE  580.x-584.x, 586, 587, 588.x-589.x |  |
|  | 52 | Other diseases of the genitourinary system | 590.x-608.x, 610.x, 611.x, 614.x-629.x |  |
| Diseases of the skin and subcutaneous tissues | 53 | Diseases of the skin and subcutaneous tissues, including No rheumatoid psoriasis | 680.x-686.x, 690.x-695.x, 696.0, 696.2-696.5, 696.8, 697.x, 698.x, 700.x-709.x, 696.1 | D05BB01, D05BB02, D05AX |
| Diseases of the musculoskeletal system and connective tissue | 54 | Autoimmune disease (Rheumatoid arthritis, Rheumatoid psoriasis, Anchylosing spondylitis, Systemic sclerosis, Systemic lupus erythematosus) | 714.0, 696.0, 720.0, 710.1x, 710.0x |  |
|  | 55 | Other diseases of the musculoskeletal system and connective tissue | 710.2-710.9, 711.x-713.x, 714.1x, 714.9x, 715.x-719.x, 720.1x-720.9x, 721.x-739.x |  |
| Symptoms, signs and ill-defined conditions | 56 | Symptoms, signs and ill-defined conditions | 780-799 |  |
| Other conditions | 57 | Transplantation | V42 | L04AA01, L04AA02, L04AA03, L04AA04, L04AA05, L04AA06, L04AA08, L04AA09, L04AA10, L04AA11, L04AA12, L04AA14, L04AA15, L04AA16, L04AA17, L04AA18, L04AA19, L04AA21, L04AD01, L04AD02, L04AX01 |
|  | 58 | Chronic pain | 338.2, 338.4 | N02AA01, N02AG01, N02AE01, N02AB03, N02AA05, N02AA55, N02AA03, N02AX06 |
|  | 59 | Corticosteroids |  | H02 |

**Supplementary figure legends**

**Figure S1**. Forest plots depicting the unadjusted association between selected features of the study cohort (citizens who completed scheduled vaccination plan with three doses) and the odds of severe COVID-19 illness.

**Figure S2**. Forest plots depicting the unadjusted association between 51 diseases/conditions the members of the study cohort (citizens who completed scheduled vaccination plan) suffered from and the odds of severe COVID-19 illness. The 51 diseases/conditions are sorted for decreasing values of the observed association strength.
